# Most German Speakers Ignore the Cue That Best Predicts Plural Class

**DOI:** 10.1162/OPMI.a.320

**Published:** 2025-12-18

**Authors:** Kate McCurdy, Timothy J. O’Donnell, Adam Lopez, Sharon Goldwater

**Affiliations:** Department of Language Science and Technology, Saarland University, Saarbrücken, Germany; Department of Linguistics, McGill University, Montreal, Quebec, Canada; Institute for Language, Cognition, and Computation, School of Informatics, University of Edinburgh, Edinburgh, UK

**Keywords:** morphology, generalization, psycholinguistics, Bayesian modeling

## Abstract

Researchers generally assume that speakers use the linguistic information available to them. For instance, if one grammatical category robustly predicts another grammatical category, we expect speakers to reproduce this conditional relationship during language production. Here, we investigate this assumption for grammatical gender in German. Gender is the single cue which most strongly predicts the plural class of existing German nouns, but behavioral studies with novel nouns have found mixed results regarding the role of gender in plural generalization. Across three experiments, we examine how individual German speakers use grammatical gender when producing plural forms of novel nouns. We find that most speakers effectively ignore gender during plural class production, even under experimental manipulations that encourage them to attend to this cue. These results point toward an underexplored direction in cognitive science: accounting for the linguistic information that speakers do not use.

## INTRODUCTION

Language production requires speakers^1^ to combine linguistic elements into novel sequences, and in so doing, select a specific combination from the vast space of possible linguistic forms. A fundamental goal of language research is to characterize this process. What sources of information are available to constrain the search for novel linguistic forms, and which of these sources do speakers consistently use?

The most direct influence comes from language itself: speakers know the distribution of forms which characterizes their language, and apply this knowledge in production. One way this happens is through conditional patterns between linguistic variables, as the presence of one specific linguistic feature (which we’ll call the *conditioning variable*) influences or even determines another (the *target variable*). For example, in languages with vowel harmony, the choice of grammatical suffix (e.g., the Turkish plural marker *-ler*/*-lar*) is typically determined by the presence of a specific vowel feature in the root word (e.g., front vowel *ip* ‘rope’ *ipler* ‘ropes’ vs. back vowel *kız* ‘girl’ *kızlar* ‘girls’). Studies of novel word formation in Turkish (Arik, 2015) and Hungarian (Hayes & Londe, 2006), inter alia, show that speakers consistently match the conditional patterns of vowel harmony observed in their languages. However, not all aspects of linguistic distributions are relevant to production, and speakers may not use the full set of distributional information which is in principle available. For instance, in another novel word study, Becker et al. (2011) find that Turkish speakers condition on certain features (e.g., word length) but not others (e.g., the preceding vowel) when producing laryngeal consonants—even though all of the phonotactic patterns in question are equally statistically prevalent in Turkish. Specifying the distributional information which is not only available but *relevant* to producing a given linguistic target variable is a core aim of both theoretical and psycholinguistic research.

Identifying linguistically relevant information can be more complicated for variables which *probabilistically* condition targets, because aggregate probabilities at the group level can obscure important variation at the individual level. On one hand, speakers may produce linguistic target variables at rates roughly corresponding to their distributional frequencies, leading to group-level probability measurements which mirror individual-level behavior. This behavior is known as frequency-matching or *probability-matching*, and has been attested in production experiments from both artificial languages (e.g., Hudson Kam & Newport, 2005, 2009) and natural languages (e.g., Hayes & Londe, 2006; Hayes et al., 2009). On the other hand, Pierrehumbert notes that “pooled data can give a spurious appearance of probability-matching in cases where different participants learn categorically different systems” (2022, p. 652); for instance, one speaker may deterministically condition a target variable on a certain feature, while another speaker may ignore that feature entirely. Studies which claim to find probability-matching must show that “statistical patterns in the output of individual learners match those in the input, which are assumed to be the same as those in the ambient language” (Pierrehumbert, 2022, p. 652). The psycholinguistic literature documents many probabilistic conditional relationships between linguistic variables, but few accounts of their realization in individual speaker production.

This paper investigates how individual speakers realize the conditional relationship between two well-studied linguistic features: the grammatical gender and plural class of German nouns. The observation that gender predicts plural class in the German noun lexicon is widely affirmed in the research and education literature. Formal linguistic theories of German plural inflection assign gender a causal, usually central, role (e.g., Augst, 1979; Bittner, 1994); computational models, including artificial neural networks, consistently learn to condition plural class upon gender (Dankers et al., 2021; Goebel & Indefrey, 2000); and learners of German as a second language are often explicitly taught to attend to noun gender when guessing an unknown plural form (Ković et al., 2008). The predictive relation appears to generalize to novel word formation as well—in a large-scale production study, Zaretsky and Lange (2016) identify grammatical gender as a statistically significant predictor of plural class, the most robust predictor in their analysis. It is not clear, however, whether this reflects consistent gender conditioning behavior by the majority of speakers, and findings on this topic have been mixed; in a similar experiment, Mugdan (1977) found that speaker plural production was generally not sensitive to noun gender. Although grammatical gender provides a clear distributional cue to the plural class of existing German nouns, its role in conditioning plural class generalization to novel nouns remains an open question.

We study how grammatical gender influences the plural class production of individual speakers across three behavioral experiments, using the same novel noun stimuli for which Zaretsky and Lange (2016) found statistically significant gender sensitivity. We use the information-theoretic quantity of *mutual information* to measure the influence of grammatical gender on plural class production for each individual participant, and compare this to the mutual information between these two variables in the German noun lexicon. The first experiment presents the novel nouns with systematically varied grammatical gender; the second adds a financial incentive to encourage more consciously targeted plural class selection; and the third asks participants to produce both the grammatical gender and plural form for each noun. In each of these experiments, we find that the median adult German speaker pays minimal or no attention to grammatical gender when forming the plural of unknown nouns—and this holds even when we account for phonological cues in post-hoc analysis.^2^ The findings presented here suggest that we are still far from understanding how human speakers use the distributional information available to them in generalizing linguistic categories. In an age when powerful computational models trained on linguistic distributions are capable of many human-like generalizations about language structure (Mahowald et al., 2024), characterizing when and how speakers *fail* to use ostensibly relevant linguistic information may open critical new avenues for scientific inquiry.

## BACKGROUND: GRAMMATICAL GENDER AND THE GERMAN PLURAL SYSTEM

Each noun in the German lexicon has two inherent lexical attributes: grammatical gender and plural class. Grammatical gender can be masculine, feminine, or neuter, and is expressed on the definite article preceding a nominative singular noun, e.g., *der Mann* “the (masc.) man”, *die Frau* “the (fem.) woman”, *das Kind* “the (neut.) child”. The plural class of a noun is characterized by how it differs from the singular form of the noun,^3^ e.g., *Mann*—*Männer*, *Frau*—*Frauen*, *Kind*—*Kinder*.^4^ Plural nouns may be marked by a suffix, an umlaut, both, or neither. For simplicity, in this work we focus only on suffixes, taking them to define five separate plural classes: *-e*, *-er*, *-(e)n*, *-s*, and the null marker ø for nouns which take no suffix. German speakers are sensitive to both gender and plural class in online language processing, with mismatched categories eliciting neural signatures of syntactic violation (e.g., Misersky et al., 2019; Regel et al., 2019).

### Formalizing Informativity

**Figure 1.**
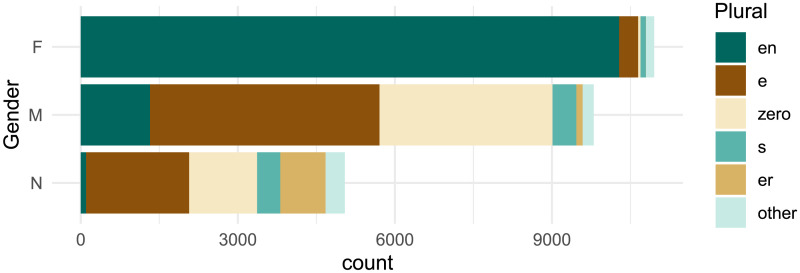
Grammatical gender and plural class of German nouns in the CELEX lexicon (Baayen et al., 1995). Plural classes are ordered by type frequency. Note the strong correlation between gender and plural class; e.g., 94% of feminine nouns take the *-en* plural suffix.

Figure 1 shows a clear distributional relationship between the grammatical gender and plural class of German nouns in the CELEX lexicon (Baayen et al., 1995). Here, and in the rest of the paper, we quantify this relationship in terms of *mutual information* as defined by information theory (Shannon, 1948). First, we define the categorical probability distribution *P* over plural classes *C*, and characterize its uncertainty in terms of Shannon entropy *H*(*C*), measured in bits:

$$H\left(C\right)=-\sum_{c\in C}\text{P}\left(c\right)\log_{2}\;\text{P}\left(c\right)$$ (1)

Calculating this for six plural classes (five described above and a catchall “other” class) using observed probabilities in CELEX gives us *H*(*C*) = 1.98 bits. To give some intuition, we can think about this roughly in terms of guessing. If we needed to guess the plural class of some random noun from the German lexicon, given no additional information, the optimal strategy would be to first guess the most probable class (*-en*), then the second (*-e*), and so on. Roughly speaking, entropy tells us the average of the lower-bound number of guesses needed under this strategy (Massey, 1994). *H*(*C*) = 1.98 implies we would need about 2 guesses on average to identify the plural class of a randomly drawn noun.

Now that we know the difficulty of blindly guessing the plural class for a random German noun, we can estimate how that uncertainty would be reduced by knowing that noun’s grammatical gender. We quantify the uncertainty of plural class *C* conditioned on grammatical gender *G* in terms of conditional entropy *H*(*C*|*G*), again using the probabilities we observe in the corpus:

$$H\left(C|G\right)=-\sum_{g\in G}\text{P}\left(g\right)\sum_{c\in C}\text{P}\left(c|g\right)\log_{2}\;\text{P}\left(c|g\right)$$ (2)

This yields *H*(*C*|*G*) = 1.31 bits. We have substantially reduced uncertainty as measured in guesses—if we know nothing about a random noun except its grammatical gender, we can optimize our strategy accordingly (i.e., initially guess *-en* for feminine nouns and *-e* for non-feminine) and require closer to one than two guesses on average. We express this uncertainty reduction as mutual information:

$$MI\left(C;G\right)=MI\left(G;C\right)=H\left(C\right)-H\left(C|G\right)$$ (3)

The result is *MI*(*C*; *G*) = 0.67 bits. If we normalize this quantity with respect to overall entropy (Miller & Nicely, 1955), we observe that roughly 34% ($\frac{0.67}{1.98}$) of plural class variation in the German noun lexicon appears to be conditioned on grammatical gender.^5^ If German speakers are sensitive to this strong predictive relationship, we might expect similar levels of mutual information between these two variables to arise when individuals produce plural forms for unknown nouns.

### Gender and Plural in Linguistic Theory

The obvious relationship between grammatical gender and plural class has informed nearly a century of theoretical linguistic analyses (Bloomfield, 1933; Elgersma & Houseman, 1999; Indefrey, 1999; Schuhmann & Putnam, 2021; Trommer, 2021; Wegener, 1994; Wiese, 1996). Some researchers have even identified grammatical gender as the primary factor structuring the German plural system (e.g., Augst, 1979; Bittner, 1994, 2000). Yang (2016, Ch. 4) provides an illustrative recent example. His analysis recursively applies a statistical decision rule to infer plural forms over a corpus of nouns drawn from child-directed speech. This process yields a grammar which applies a gender-conditioned rule (*gender* = *FEMININE* → *plural* = *-en*) as its first step, followed by several phonologically-conditioned rules, and concludes with a second gender-conditioned rule (*gender* ≠ *FEMININE* → *plural* = *-e*) as the final step in the derivation. Like many other theories, this account assigns grammatical gender a primary and direct causal influence on plural class production.

### Gender and Plural in Computational Models

Researchers have consistently found that general statistical learners such as neural networks treat grammatical gender as highly informative to plural class—which is unsurprising, given the high mutual information between these two variables. Goebel and Indefrey (2000) train a simple recurrent neural network on German plural inflection and report that the model learns gender-dependent patterns of plural class prediction. Two decades later, Dankers et al. (2021) reproduce this result with modern long-short-term-memory networks; their analysis of training dynamics finds that the first rule identified by Yang’s grammar (i.e., *gender* = *FEMININE* → *plural* = *-en*) also describes the first robust generalization learned by the neural model. Finally, Beser (2021) confirms that modern Transformer models learn the same gender-conditioned approach to processing German plural inflection. Both theoretical and computational approaches agree: grammatical gender is a highly informative cue to plural class, and speakers should use this information when inflecting unknown German nouns. We build on this computational literature later in our analysis by training recurrent neural networks (RNNs) on the task of plural production, and comparing their predictions with speaker behavior.

### Gender and Plural in Behavioral Experiments

Given the seemingly uncontroversial role of gender in theoretical and computational studies of the German plural, behavioral evidence is surprisingly sparse and inconclusive. Several novel word experiments on German plural generalization mention, but do not statistically analyze, effects of grammatical gender (Gawlitzek-Maiwald, 1994; Köpcke, 1988; Marcus et al., 1995; Spreng, 2004). Mugdan (1977) highlights the surprising *absence* of gender effects in his study, which was apparently even brought to his attention by participants:

Es scheint, daβ es vor allem der Wortklang war, der die Vpn bei der Auswahl eines Pluralallomorphs leitete, während insbesondere das Genus offenbar weitgehend unbeachtet blieb. (Das wurde auch von manchen Vpn nach Abschluβ des Tests erwähnt.) (1977, p. 172)

*It seems that the participants’ selection of plural allomorphs was guided above all by the sound of the word, while gender in particular remained largely ignored. (Several participants even mentioned this at the end of the test.)*^6^

In this quote, Mugdan suggests that phonology exerts a stronger effect on plural class production than grammatical gender. This idea has been developed in subsequent research; for instance, Spreng (2004) proposes a cue hierarchy in which phonology *mediates* the influence of gender on plural class, and Plag et al. (2023) use segmental phonology to model plural behavior in aphasic patients.

On the other hand, in a large-scale study using the stimuli developed by Marcus et al. (1995), Zaretsky and Lange (2016) find that grammatical gender is the most reliable and statistically significant factor predicting participants’ plural generalization. In light of grammatical gender’s importance in the theoretical and computational literature on German plural classes, the discrepant behavioral findings discussed here motivate our experiments and fine-grained analysis.

### Summary and Motivation

The data and literature reviewed here clearly show that the distribution of plural classes of German nouns is closely tied to the distribution of grammatical gender classes over those same nouns. In other words, if a German speaker doesn’t know the plural class for a given noun, grammatical gender should provide a highly informative cue—indeed, according to many linguistic theories and computational models, gender should be *the* most influential predictor of plural class. Existing behavioral studies, however, do not clearly indicate whether speakers use this distributional information as expected. In particular, while Zaretsky and Lange (2016) found a statistically significant effect of grammatical gender on plural class production in the aggregate, we do not know whether this reflects the behavior of most German speakers. This gap in the literature motivates the series of experiments presented here. We use the monosyllabic novel noun stimuli developed by Marcus et al. (1995) (Table 1), following Zaretsky and Lange and other previous work (e.g., Goebel & Indefrey, 2000; Hahn & Nakisa, 2000; McCurdy et al., 2020), to investigate how grammatical gender affects the plural class productions of individual speakers.

**Table 1.** Experimental novel noun stimuli, originally developed by Marcus et al. (1995).

Bral	Nuhl	Raun	Bnaupf	Fneik	Pleik
Kach	Pind	Spand	Bneik	Fnöhk	Pnähf
Klot	Pisch	Spert	Bnöhk	Pläk	Pröng
Mur	Pund	Vag	Fnahf	Plaupf	Snauk

They are divided into Rhymes (phonologically similar to existing German nouns) on the left and Non-Rhymes (phonologically atypical) on the right, though this distinction is not relevant to the current experiments.

## METHODS

### Hypotheses

Suppose that an individual German speaker uses grammatical gender as a cue to predict the plural class of unknown nouns, based on the distributional information contained in the German noun lexicon. If that speaker produces plural forms for a sample of unknown nouns, and we measure the mutual information between gender *G* and plural class *C* (*MI*(*C*; *G*); Equation 3) for that speaker’s productions, we would expect to see roughly the amount of *MI*(*C*; *G*) that we observe in the lexicon as a whole, with some allowance for noise. Note that this expectation is agnostic with respect to underlying representations or procedures. A wide range of processes may give rise to the observed conditional relationship between grammatical gender and plural class in the German noun lexicon. Here, we assume only that this conditional distribution influences speaker productions. This influence could occur directly, meaning that speakers match the plural class probabilities observed in the lexical distribution; indirectly, meaning that some cognitive or linguistic process both drives speaker behavior and produces the observed lexical distribution; or some combination of the two. In any case, all of these scenarios should produce similar outcomes: speakers’ plural productions should reflect roughly the level of gender conditioning that we see in the German noun lexicon *overall*. This is our hypothesis *H*_1_:

$$H_{1}:MI\left(C;G\right)\approx {MI}_{\text{overall}}$$ (4)

Alternatively, if a speaker does not use grammatical gender to predict plural class, then we would expect their productions to reflect a lower value of *MI*(*C*; *G*). In principle, the *MI* in this case should be zero; in practice, however, mutual information is a non-negative quantity and some positive *MI* values will arise by chance. We expect, however, that if *C* and *G* have fully *independent* distributions in a speaker’s productions, the noisy *MI*_*independent*_ will be consistently lower than the observed *MI*_*overall*_. This noise estimation forms our null hypothesis *H*_0_:

$$H_{0}:MI\left(C;G\right)\approx {MI}_{\text{independent}}$$ (5)

We evaluate these hypotheses^7^ using a three-step procedure. First, we generate a simulated data set for each hypothesis, showing how this behavior would be realized under our experimental protocol. Second, we use the simulated data to fit a Bayesian model corresponding to each hypothesis. Finally, we use our experimental protocol to gather behavioral data, and estimate its likelihood under these two models to evaluate which hypothesis provides the better fit.

### Simulation

Our experimental design measures gender conditioning on the level of individual speakers. To simulate speaker behavior, we rely upon one specific aspect of that design: the number of items presented with each gender. Given 24 items (Table 1), our initial experiment presents every participant with 8 feminine *F*, 8 masculine *M*, and 8 neuter *N* nouns. We simulate predicted plural class patterns under *H*_0_ and *H*_1_.

We generate the simulated data by sampling from the multinomial distribution corresponding to each hypothesis, using probabilities derived from observed lexical counts (Table 2). For each simulated participant, we sample 8 plural classes *C* separately for each grammatical gender. We simulate *H*_1_ using gender-conditioned probabilities:

$$\begin{array}{l} C_{F}^{H_{1}}∼\text{Multinomial}\left(8,p_{F}\right)\\ C_{M}^{H_{1}}∼\text{Multinomial}\left(8,p_{M}\right)\\ C_{N}^{H_{1}}∼\text{Multinomial}\left(8,p_{N}\right) \end{array}$$ (6)

Conversely, we simulate the null hypothesis *H*_0_ without any assumption of gender conditioning. For each gender category, we sample from *p*_*All*_, the distribution over all plural classes in the lexicon:

$$\begin{array}{l} C_{F}^{H_{0}}∼\text{Multinomial}\left(8,p_{\text{All}}\right)\\ C_{M}^{H_{0}}∼\text{Multinomial}\left(8,p_{\text{All}}\right)\\ C_{N}^{H_{0}}∼\text{Multinomial}\left(8,p_{\text{All}}\right) \end{array}$$ (7)

For each hypothesis, we simulate 10,000 participants, and calculate the entropy *H*(*C*) and mutual information *MI*(*C*; *G*) for each simulated participant (cf. Ferdinand et al., 2019).

**Table 2.** Plural class percentages per grammatical gender and over all genders, calculated from CELEX (cf. Figure 1).

Hypothesis	Gender	*-en*	*-e*	ø	*-s*	*-er*	Other
*H*_1_	*F*	94	3	0	1	0	1
	*M*	13	45	34	5	1	2
	*N*	2	39	26	9	17	7
*H*_0_	All	45	26	18	4	4	3

Each row sums to 100%. These observed distributions define the probability vectors *p*_*F*_, *p*_*M*_, *p*_*N*_, and *p*_*All*_ used for data simulation.

### Model

The goal of our model is to quantify the plausible range of gender conditioning values *MI*(*C*; *G*) we might observe under our competing hypotheses *H*_0_ and *H*_1_. Our choice of model is informed by certain assumptions and constraints. The main constraint is that, by definition (cf. Equation 3), gender conditioning *MI* highly depends upon overall plural class entropy *H*(*C*). Intuitively, we can imagine a speaker who always produces the same plural class for each word, yielding a distribution with *H*(*C*) = 0. In this case, we also set *MI*(*C*; *G*) = 0: if there is no variation in plural class production, then none of it can be conditioned on gender. Conversely, if a speaker produces a large variety of plural classes such that *H*(*C*) is high, then grammatical gender could potentially condition a large portion of that variation.

Our model reflects the dependence of *MI* upon *H* in two ways. First, we normalize *MI*(*C*; *G*) to represent a fraction of *H*(*C*), and therefore take $\frac{MI}{H}$ as our dependent variable. Second, beyond normalization, we also model $\frac{MI}{H}$ as a function of *H*(*C*) in order to further contrast our two hypotheses *H*_0_ and *H*_1_. Under *H*_0_, *MI*_*independent*_ is simply noise. As *H*(*C*) grows, we expect that *MI*_*independent*_ will grow too, as more variation is available to be “explained” by gender, even though the “explanation” is random noise. Under *H*_1_, we expect the opposite: the structured conditional relation in *MI*_*overall*_ will be obscured by random noise, therefore it should decrease as variation increases in *H*(*C*).

We specify a Bayesian regression model for hypothesis testing, using the brms library (Bürkner, 2017) library in R (R Core Team, 2023). As discussed, we want to model *MI*(*C*; *G*) appropriately as a quantity bounded between zero and *H*(*C*). The Beta distribution can model values between the range of zero and one, which we achieve by normalizing *MI*. For each participant *i*:

$${\frac{MI}{H}}_{i}∼\text{Beta}\left(\mu_{i},\varphi \right)$$ (8)

We model *μ*_*i*_ using linear predictors and the logit link function. Our linear predictors are the intercept and the overall plural class entropy *H*, motivated by the reasons discussed above.

$$\begin{array}{l} \mu_{i}={\text{logit}}^{-1}\left(\beta_{0}+\beta_{1}H_{i}\right)\\ {\text{logit}}^{-1}\left(x\right)=1/\left(1+e^{-x}\right) \end{array}$$ (9)

As the Beta distribution is sensitive to weak priors, we use our simulated data for calibration. Predictive checks showed that the default brms prior values yielded implausibly overdispersed prior distributions, with extreme concentrations of probability mass around zero and one. We iteratively refined our priors until the predictive distribution roughly matched the scale and shape of the simulated data while maintaining sufficient uncertainty over the full range of possible values.

$$\begin{array}{l} \beta_{0}∼\text{Normal}\left(-0.5,0.5\right)\\ \beta_{1}∼\text{Normal}\left(0,0.1\right)\\ \varphi ∼\text{Gamma}\left(5,1\right) \end{array}$$ (10)

We use this model specification to fit two separate models on the simulated data for each hypothesis, and verify their quality with posterior predictive checks. Table 3 shows the resulting learned parameters. We apply these models for hypothesis evaluation on the experimental data.

**Table 3.** Posterior estimates for coefficients of two Bayesian regression models predicting *MI*(*C*; *G*)/*H*(*C*)—mutual information between plural class *C* and gender *G*, normalized by plural class entropy *H*(*C*)—under *H*_0_ (gender conditioning at chance levels) and *H*_1_ (gender conditioning as seen in the German noun lexicon).

	*H*_0_				*H*_1_			
	Estimate	Est. Error	l-95% CI	u-95% CI	Estimate	Est. Error	l-95% CI	u-95% CI
Intercept	−2.58	0.01	−2.60	−2.55	0.34	0.01	0.32	0.36
*H*(*C*)	0.46	0.01	0.45	0.47	−0.30	0.01	−0.31	−0.29
*φ*	29.74	0.13	29.48	30.00	39.87	0.18	39.52	40.21

The models are trained on simulated datasets *C*^*H*0^ and *C*^*H*1^ respectively.

## EXPERIMENT 1

### Stimuli

We use the 24 novel nouns developed by Marcus et al. (1995) (Table 1). In their original study, Marcus et al. did not find any interactions between grammatical gender and other predictors, and did not report any analysis for main effects of gender; however, Zaretsky and Lange (2016) report gender effects in the expected direction on those same stimuli—participants used *-en* more on feminine nouns, and *-e* more for nonfeminine nouns. Zaretsky and Lange speculate that these discrepant findings stem from differences in the two study designs: scale (the earlier study had 48 participants, the later one 585) and task (acceptability ratings vs. elicited productions). A third differentiating factor is the presence of additional semantic cues in the Marcus et al. study, which provided sentence contexts around the novel nouns; for example, a sentence like *Die grünen BRALS sind billiger* (“The green brals are cheaper”) would imply that the unknown word *Bral* referred to an object, whereas *Die BRALS sind ein bißchen komisch* (“The Brals are a bit weird”) would imply that *Bral* was a family name. It’s possible that this manipulation directed participant focus toward semantic cues rather than grammatical gender. Zaretsky and Lange provided no semantic context in their experiment, only presenting the indefinite article and word form to participants (e.g., *Ein Bral*, “a [masculine/neuter] bral”). Our experimental design is closer to that of Zaretsky and Lange (2016): we elicit plural form productions and provide no semantic cues. This suggests we might also expect to find a robust effect of grammatical gender for these stimuli.

### Participants and Procedure

**Figure 2.**
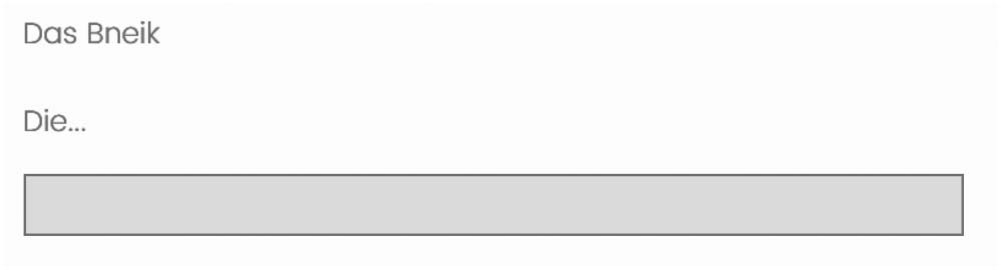
Stimulus item presentation for Experiment 1.

We collected production data from 92 native German speakers^8^ through an online survey. After providing consent, participants completed an onboarding task in which they had to provide the plural form for 12 real German nouns, before proceeding to the 24 experimental stimuli. Participants saw each noun in the singular with a definite article indicating grammatical gender (e.g., *Der Bral* for masculine, *Das Bral* neuter, *Die Bral* feminine), and typed the corresponding plural-inflected form (cf. Figure 2). Participants were randomly assigned to one of three lists. Grammatical gender was counterbalanced within lists (each participant saw 8 feminine, 8 masculine, and 8 neuter nouns) and across lists (each noun appeared with a different gender in each list). Noun presentation order was randomized.

### Results

**Figure 3.**
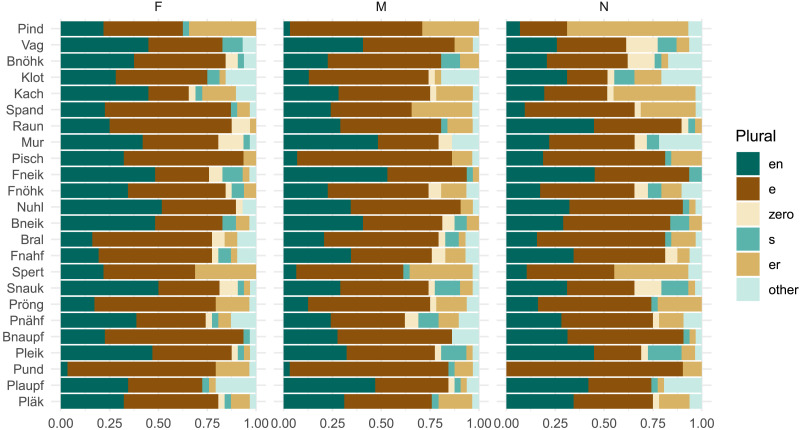
Per-item plural distribution results in Experiment 1, ordered by gender conditioning from *MI*(*C*; *G*) = 0.18 bits (*Pind*) to 0.04 bits (*Pläk*).

**Figure 4.**
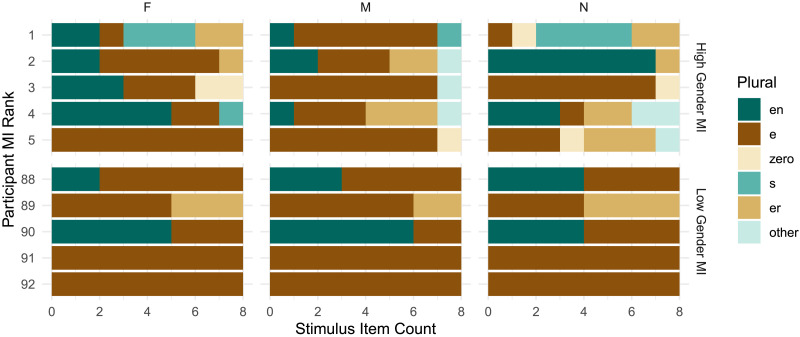
Individual participants in Experiment 1 with the highest (top) and lowest (bottom) observed gender conditioning. Each row displays an individual participant’s plural productions for feminine (F), masculine (M), and neuter (N) nouns, with row number indicating their rank ordered by *MI*(*C*; *G*).

We start with a more traditional assessment of statistical significance. Our findings reproduce the grammatical gender effect found by Zaretsky and Lange (2016). We follow their analysis procedure and fit separate binomial mixed-effects models^9^ to predict the occurrence of the two most frequent plural classes, *-en* and *-e*, in our experimental data. We use only grammatical gender as a fixed effect predictor,^10^ and fit random intercepts for items and participants. These statistical analyses reproduce the expected effects: participants are significantly more likely to produce the *-en* plural for nouns with feminine gender (*β* = 0.51, *se* = 0.14, *z* = 3.73, *p* < 0.001), and the *-e* plural for nouns with masculine gender (*β* = 0.27, *se* = 0.12, *z* = 2.21, *p* < 0.03). This statistical result is fairly robust; if we assume a stronger random effects structure with per-participant random slopes for gender,^11^ we still see a significant increase for *-en* with feminine nouns (*β* = 0.65, *se* = 0.17, *z* = 3.73, *p* < 0.001), although the *-e* increase for masculine nouns is only marginally significant (*β* = 0.24, *se* = 0.12, *z* = 1.94, *p* < 0.1). If we visually examine the item-level results (Figure 3), however, we see that the size of the effect is small, in that the columns divided by gender do not look that different from each other; it is unclear whether this statistically significant effect reflects systematic gender-conditioning behavior by participants. Figure 4 shows productions from individual participants with the highest and lowest levels of gender conditioning.

**Figure 5.**
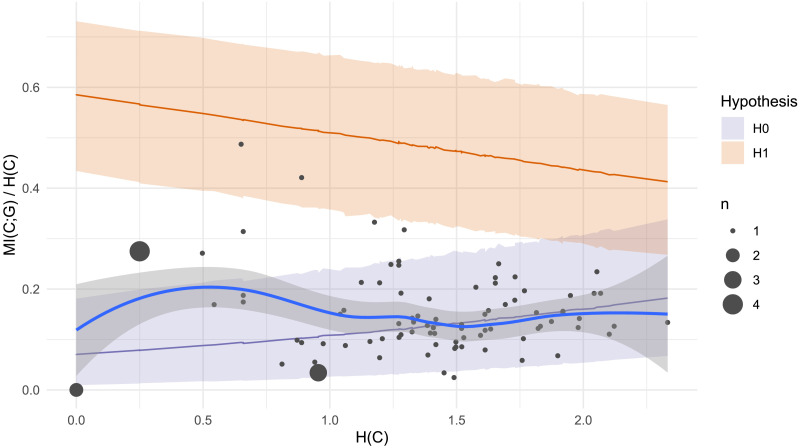
Plural class entropy *H*(*C*) and mutual information with grammatical gender *MI*(*C*; *G*) for each individual participant (dot) in Experiment 1. Larger dots represent multiple participants. Shaded areas represent 95% confidence intervals under a) Bayesian regression models for *H*_1_ (gender conditioning as expected under the lexicon; orange) and *H*_0_ (null hypothesis of chance-level gender conditioning; purple) and b) a non-parametric Loess smoothed fit to the participant data (grey with blue line). Out of 92 participants, only 5 have a log Bayes Factor greater than 0, indicating more compatibility with *H*_1_; the other 87 show gender conditioning levels which are more likely under the null hypothesis *H*_0_.

For a more fine-grained analysis, we calculate the likelihood of individual participant productions under the Bayesian regression models described in the previous section. Figure 5 plots individual participants and the models’ predictions. Of 92 participants in the study, only five (i.e., the five participants in the top panel of Figure 4) have a positive log odds ratio, indicating gender conditioning levels compatible with *H*_1_. The plural productions of the other 87 participants have negative log Bayes Factors, indicating higher likelihood under the null hypothesis *H*_0_. When we sum log likelihood over all participants to compute an overall Bayes Factor, the result is 0, indicating strong evidence for *H*_0_. Although our mixed effect model analysis found a statistically significant effect of grammatical gender on plural class production in our experiment overall, this is driven by only a small number of speakers. 95% of our study participants appear to largely ignore grammatical gender even though it is highly informative to plural class.

## EXPERIMENT 2

The main finding of Experiment 1 is that most adult speakers of German do not match *MI*_*overall*_, the level of gender conditioning observed in the German noun lexicon overall. In other words, they do not seem to view grammatical gender as a particularly informative cue to the plural class of unknown nouns in our experiment. This is surprising, because grammatical gender predicts plural class in the German noun lexicon (cf. Figure 1), and a large body of theoretical and computational literature has identified gender as the most important cue to plural class generalization.

One possible explanation is a design flaw in our behavioral experiment. We see high variability in the production data for both items (Figure 3) and speakers (Figure 4). Perhaps speakers simply had no compelling reason to reduce their uncertainty over plural classes while completing the study, and therefore did not make maximal use of the information available to them, i.e., noun gender. In Experiment 2, we try to address this limitation by adding a financial incentive structure.

In an interactive artificial language learning study, Perfors (2016) found that financial incentives could systematically affect adult speakers’ probability-matching behavior. Participants operated in pairs, and learned to map words to specific objects. In one condition, both participants in a given pair were compensated based on the *accuracy* of the mappings they learned, evaluated by an objective standard; participants in this condition tended to probability-match. Other participants, however, were compensated instead based on how closely they *matched* each others’ learned mappings. In this condition, participants were significantly more likely to *regularize*, i.e., produce the most likely word. Perfors notes that this result aligns with earlier findings of financial incentives leading participants to reduce probability-matching behavior (Shanks et al., 2002; Vulkan, 2000).

Based on this finding, we extend the design of Experiment 1 to include financial incentives for participants to match others’ responses. We hypothesize that this will motivate speakers to draw on all available information in predicting the most likely plural form for each noun. Our experimental presentation provides very limited information: speakers see only the grammatical gender and phonological form of the singular noun, with no additional semantic or sentence context. Given the high statistical informativity of grammatical gender and the limited information available, we expect a substantially higher proportion of mutual information between grammatical gender and plural class in speaker productions compared to Experiment 1.

Experiment 2 uses the same design and materials as Experiment 1, with one additional manipulation: participants were offered financial incentives for producing the same plural form as the majority of other participants. Table 4 shows the updated instructions. While two cents may not appear a significant financial incentive, a participant who produced the majority plural form each time would earn an additional 48 cents—which, given the short amount of time spent on the study (median time to completion was roughly 7 minutes), would represent a 35% increase in their earnings for the study.

**Table 4.** German-language instructions for Experiment 2 and their English translation.

In dieser Studie werden Sie statt echten Wörtern (wie z.B. “Messer”) fiktive Wörter sehen, die andere Teilnehmer auch schon gesehen haben. Für jedes Wort, **vermuten Sie bitte, welche Pluralform am häufigsten von anderen Teilnehmern angegeben wurde**. Bei jeder richtiger Vermutung verdienen Sie noch 2 Cent zusätzlich als Bonus.

In this study, rather than real words such as “knife,” you will see made-up words which have already been seen by other participants. For each word, **please guess which plural form was given most frequently by other participants**. You will receive 2 cents additionally as a bonus for each correct guess.

Note that this design is not directly parallel to the experiments considered above, where participants typically receive direct feedback on their responses; for instance, the participants in Perfors’ match condition could observe how well their productions aligned with those of their partner. By contrast, speakers in our experiment receive no real-time feedback about others’ behavior. The only reward signal comes in the form of the bonus, which is calculated and distributed after the experiment. Nonetheless, we reason that speakers may be motivated to use any available information to increase their reward. Given the strong statistical relationship between grammatical gender and plural class, they might reason that *other* speakers condition their responses on the information available to them—i.e., grammatical gender—and therefore attend more reliably to this cue. 100 German speaking participants were recruited on Prolific for this study. Participants who had taken part in Experiment 1 were excluded.

### Results

**Figure 6.**
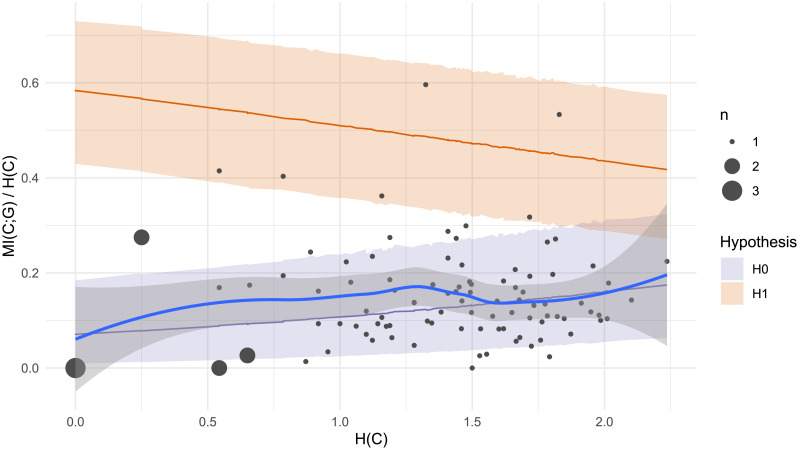
Plural class entropy *H*(*C*) and mutual information with grammatical gender *MI*(*C*; *G*) for each individual participant (dot) in Experiment 2. Larger dots represent multiple participants. Shaded areas represent 95% confidence intervals under a) Bayesian regression models for *H*_1_ (gender conditioning as expected under the lexicon; orange) and *H*_0_ (null hypothesis of chance-level gender conditioning; purple) and b) a non-parametric Loess smoothed fit to the participant data (grey with blue line). While two participants show super-lexical gender conditioning (above the orange line), the vast majority (93%) show behavior more consistent with the null hypothesis *H*_0_.

Our financial incentives may have successfully motivated two participants, who show super-lexical levels of gender conditioning (Figure 6). This suggests that the strong statistical connection between gender and plural class is in principle consciously available to at least some German speakers, and under the right circumstances, they can use this information to reduce uncertainty in plural class generalization. For most participants, however, our experiment did not provide these circumstances. 93% have negative log Bayes Factors, indicating more compatibility with the null hypothesis *H*_0_, and the overall Bayes Factor for the experiment is still 0.

The incentives also appear to have had no effect on overall variability. Participants in Experiment 2 produced a similarly diverse range of plural classes to Experiment 1, considered both by subjects (mean per-subject *H*(*C*): 1.34 in Experiment 1, 1.35 in Experiment 2) and by items (mean per-item *H*(*C*): 1.75 in Experiment 1, 1.78 in Experiment 2).

## EXPERIMENT 3

The previous two experiments found that most adult German speakers do not treat grammatical gender as an informative cue to the plural class of unknown nouns, and financial incentives to maximize predictability largely do not change this behavior. It is still possible, however, that these null findings reflect flawed experimental designs: perhaps the financial incentives were inadequate to motivate participants’ attention. In Experiment 3, we address this possibility with a modified experimental design in which participants assign both a plural form *and* a grammatical gender to each stimulus item. This compels participants to explicitly attend to grammatical gender, as it is directly incorporated into the task. With this experiment, we aim to rule out a specific possibility: perhaps participants in Experiments 1 and 2 simply did not attend to gender—leading to the success of *H*_0_ over *H*_1_—but *would* condition plural class on gender if forced to attend to this variable. In Experiment 3, we compel attention by asking German speakers to produce both the gender *and* the plural class for each novel noun.

### Updating Hypotheses

This experimental design changes the nature of the task: participants must produce two linguistic variables—grammatical gender and plural form—where before they only produced one. We update our computational hypotheses accordingly.

#### Simulation.

To simulate participant behavior in this experiment, we extend the method described previously to additionally sample grammatical gender. For the 24 noun stimuli, we sample grammatical gender values based on their CELEX frequency (Table 5):

$$G_{F},G_{M},G_{N}∼\text{Multinomial}\left(24,p_{G}\right)$$ (11)

where *G*_*F*_, *G*_*M*_, and *G*_*N*_ are the sampled counts for each gender.

**Table 5.** Grammatical gender frequencies calculated from CELEX (cf. Figure 1).

Gender	F	M	N
*p*_*G*_	42	38	20

We then sample plural classes as described in the Methods section, using the plural class probabilities in Table 2. For our null hypothesis *H*_0_, we assume plural class is fully independent, so we independently sample *C* per Equation 7. For the gender conditioning hypothesis *H*_1_, we modify Equation 6 to use sampled counts for each gender category:

$$\begin{array}{l} C_{F}^{H_{1}}∼\text{Multinomial}\left(G_{F},p_{F}\right)\\ C_{M}^{H_{1}}∼\text{Multinomial}\left(G_{M},p_{M}\right)\\ C_{N}^{H_{1}}∼\text{Multinomial}\left(G_{N},p_{N}\right) \end{array}$$ (12)

#### Model.

We follow the same model fitting procedure described in the Methods section, using the same specification and prior. Table 6 gives the resulting posterior estimates.

**Table 6.** Posterior estimates for Bayesian regression models predicting *MI*(*C*; *G*)/*H*(*C*)—mutual information between plural class *C* and gender *G*, normalized by plural class entropy *H*(*C*)—under *H*_0_ (gender conditioning at chance levels) and *H*_1_ (gender conditioning as seen in the German noun lexicon).

	*H*_0_				*H*_1_			
	Estimate	Est. Error	l-95% CI	u-95% CI	Estimate	Est. Error	l-95% CI	u-95% CI
Intercept	−2.47	0.01	−2.50	−2.45	1.02	0.01	1.00	1.04
*H*(*C*)	0.38	0.01	0.36	0.39	−0.62	0.01	−0.63	−0.61
*φ*	32.93	0.15	32.64	33.22	22.65	0.10	22.46	22.85

The models are trained on simulated datasets *C*^*H*0^ and *C*^*H*1^ respectively.

### Participants and Procedure

**Figure 7.**
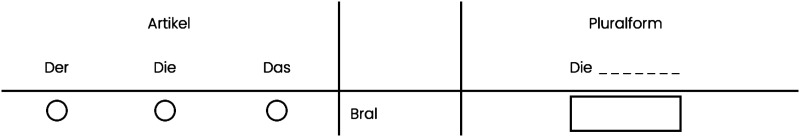
Example stimulus from Experiment 3. Given the singular form of a novel noun, participants must select one of the three grammatical gender categories on the left, and type a plural inflected form on the right.

The procedure mainly followed Experiments 1 and 2, with some minor differences. After providing consent, participants completed an onboarding task, in which they had to provide the gender and plural form for 12 real German nouns, following the instructions in Table 7. Participants had to answer these questions correctly to proceed to the experiment. After the onboarding, participants were randomly assigned to one of three lists counterbalanced for presentation order of gender. Within each list, the 24 test items were presented in randomized order. We recruited 120 speakers with German as a first language to complete an online survey using the platform Prolific. Speakers were compensated at the rate-adjusted equivalent of 11.50 USD per hour. Participants in Experiments 1 and 2 were ineligible for this study. Figure 7 presents an example stimulus item.

**Table 7.** German-language instructions for Experiment 3 and their English translation.

Für jedes der folgenden Wörter, bitte erstmal das passende Artikel auswählen. Schreiben Sie dann bitte, wie die folgenden Wörter in der Pluralform geschrieben werden sollten.

For each of the following words, please first select the appropriate article. Then write how the following words should be written in the plural form.

### Results

**Figure 8.**
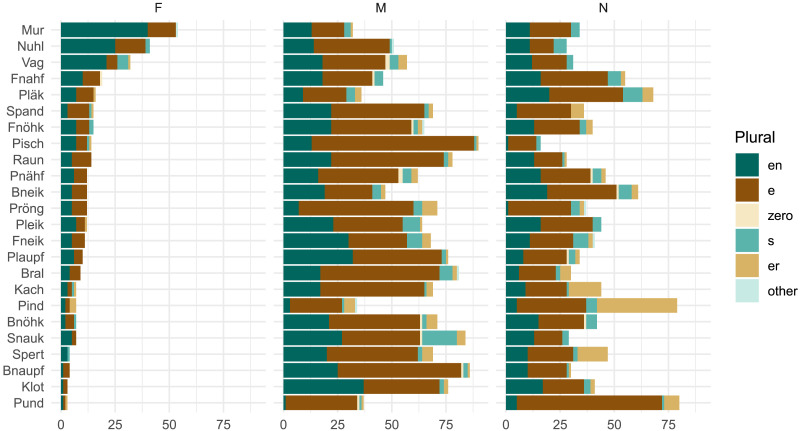
Distribution of gender and plural class assignments per stimulus item in Experiment 3. Items are ordered by decreasing number of feminine gender assignments. Participants favor non-feminine genders for these noun stimuli, but we don’t observe an increase in gender conditioning relative to previous experiments (cf. Figure 3).

We first consider participants’ assignments of grammatical gender, which diverge markedly from the overall lexical distribution of gender. While feminine nouns hold a slight plurality in CELEX (42%; cf. Table 5), the noun stimuli in our experiment are marked as feminine in only 11% of cases overall. Figure 8 shows that this varies substantially by item; for instance, 45% of participants designate *Mur* as feminine, perhaps influenced by the frequent feminine noun *Spur* ‘trace, track.’ *Mur* is the most ‘feminine’ noun and also shows the highest level of gender conditioning, with *MI*(*C*; *G*) = 0.16 bits. This remains, however, well below the 0.67 bits observed in the lexicon, i.e., *MI*_*overall*_.

**Figure 9.**
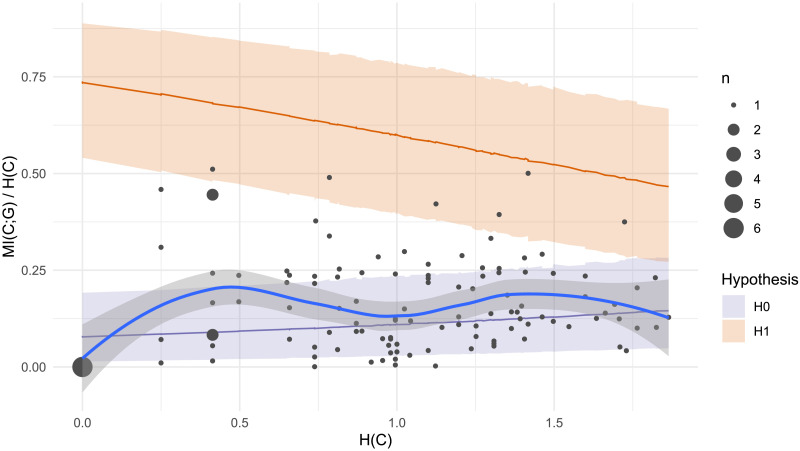
Plural class entropy *H*(*C*) and mutual information with grammatical gender *MI*(*C*; *G*) for each individual participant (dot) in Experiment 3. Larger dots represent multiple participants. Shaded areas represent 95% confidence intervals under a) Bayesian regression models for *H*_1_ (gender conditioning as expected under the lexicon; orange) and *H*_0_ (null hypothesis of chance-level gender conditioning; purple) and b) a non-parametric Loess smoothed fit to the participant data (grey with blue line). Although this task demands explicit attention to both grammatical gender and plural class, 90% of participants behave more consistently with the null hypothesis *H*_0_.

Turning to plural class, we find that our experimental intervention of compulsory gender selection has relatively little effect on subject-level gender conditioning behavior (Figure 9). The overall Bayes Factor calculation remains zero, indicating strong aggregate support for the null hypothesis *H*_0_ of no gender conditioning. If we look at individuals, the story is slightly more nuanced: 12 of the 120 participants in this experiment have log Bayes Factors greater than zero, meaning greater compatibility with *H*_1_ than *H*_0_. This proportion (10%) is larger compared to the two previous experiments (5% and 7% respectively). Our experimental manipulation may have pushed several more speakers to attend to the relationship between grammatical gender and plural class. Nonetheless, the key result here is that 90% of participants did not attend to this relationship, even in a task where they had to produce gender and plural class jointly for each noun.

## POST-HOC PHONOLOGICAL ANALYSIS

So far, we have considered only two hypotheses regarding the influence of grammatical gender on German speakers’ plural production. Under *H*_1_, speakers match *MI*_*overall*_, the level of gender conditioning observed in the lexicon as a whole; under *H*_0_, speakers instead match *MI*_*independent*_, a distribution consistent with ignoring grammatical gender in plural class production. Across three experiments, we see that *H*_0_ better predicts behavior compared to *H*_1_ for the vast majority of speakers.

This narrow contrast between *H*_0_ and *H*_1_ is motivated by the theoretical and computational literature reviewed in the Background, which identifies grammatical gender as the strongest cue to plural class membership for German nouns. Gender, however, is far from the only cue to plural class; the narrow contrast between *H*_0_ and *H*_1_ may obscure more nuanced interaction between gender and other factors. In particular, the influence of *phonology* on plural class production is well-documented (e.g., Köpcke, 1988).

Here, we re-analyze our behavioral data with a range of modeling approaches to investigate the role of phonological cues.^12^ We first formulate a third hypothesis (*H*_2_) in which gender’s influence is mediated by word shape, and repeat the simulation procedure to fit corresponding Bayesian models for *MI*_*mediated*_. Second, we assess gender conditioning levels in the orthographic neighborhoods of the stimuli. Finally, we introduce an alternative computational model of the lexicon, a recurrent neural network with the capacity to represent orthographic sequences. We use this model to implement new versions of *H*_0_ (trained without grammatical gender) and *H*_0_/*H*_1_/*H*_2_ (trained with grammatical gender), and compare how well they predict our participants’ behavior.

### The Mediation Hypothesis

Our previous hypotheses represented two experimental scenarios for German plural class production—*H*_1_, in which speakers match the *overall* level of gender conditioning in the German noun lexicon, and *H*_0_, in which speakers treat plural class as entirely *independent* from gender. We here introduce a third hypothesis, *H*_2_. In this scenario, speakers condition plural production on grammatical gender, but this conditioning is *mediated* by phonology. (Spreng, 2004 proposes a similar cue hierarchy.) Under *H*_2_, gender exerts a second-order influence on plural class, rather than the first-order influence modeled in *H*_0_. As with the previous hypotheses, we express *H*_2_ in terms of mutual information:

$$H_{2}:MI\left[C;G\right]\approx {MI}_{\text{mediated}}$$ (13)

#### Mediatory Monosyllables.

Compared to grammatical gender, phonology is a much richer source of information. Phonological cues to plural class are potentially complex and interactive. In the interest of tractable and interpretable results, we need a highly simplified phonological representation in order to formalize *H*_2_.

**Figure 10.**
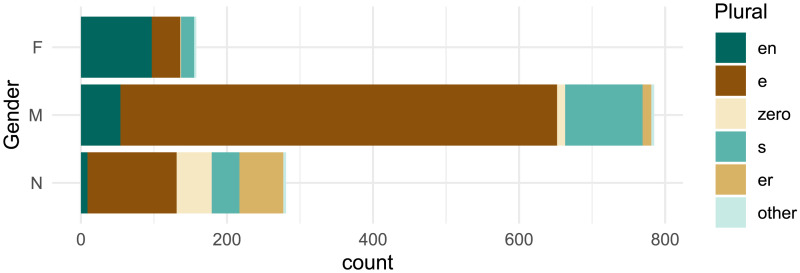
Distribution of grammatical gender and plural class for *only monosyllabic nouns* in CELEX (Baayen et al., 1995). Compare to Figure 1.

We focus our analysis on a key property of our stimuli (Table 1): they are all *monosyllabic*. Monosyllabic nouns are known to show more plural class variation compared to longer multisyllabic nouns, both in lexical analysis (Gaeta, 2008) and behavioral experiments (Köpcke, 1988). Indeed, Marcus et al. (1995) deliberately developed these stimuli to echo “irregular” existing nouns with this syllable structure. Monosyllabic nouns also diverge from the overall lexicon in terms of gender distribution, with a large majority taking masculine gender; participants’ gender assignments in Experiment 3 (Figure 8) look closer to the gender distribution over monosyllables (Figure 10) than the lexicon as a whole (Figure 1). With more variation in plural class and less diversity in gender, the relationship between grammatical gender and plural class is less statistically robust for monosyllables compared to other nouns—gender predicts roughly 17% of plural class variation for monosyllables, meaning gender loses half of its predictive power relative to the full noun lexicon (Table 8). In principle, speakers could assume that grammatical gender is equally predictive for these nouns as for the overall lexicon, i.e., match *MI*_*overall*_. If, instead, gender is subordinated to phonology, then *MI*_*mediated*_ may better characterize their plural class production behavior in our experiments.

**Table 8.** Information-theoretic characterization of plural class variation and gender conditioning in monosyllabic nouns compared to the overall lexicon.

	*H*(*C*)	*MI*(*C*; *G*)	$\frac{MI}{H}$
Overall	1.9	0.67	0.34
Monosyllables	1.71	0.30	0.17

#### Procedure.

We follow the same modeling procedure as for *H*_1_, except with lexical probabilities based only on monosyllabic nouns (Table 9). To model Experiments 1 and 2, in which grammatical gender is uniformly presented, we assume uniform gender when sampling plural class; to model Experiment 3, we first sample gender, and then plural class conditional on gender. We then fit two Bayesian regression models on the resulting simulated data, using the same formula (Equation 8) and prior (Equation 10). Table 10 provides the resulting learned parameters.

**Table 9.** Percentage of (center) plural classes within each gender *p*_*F*_, *p*_*M*_, and *p*_*N*_; and (right) total marginal percentage *p*_*G*_ of each gender for only monosyllabic nouns (Figure 10).

Gender	en	e	zero	s	er	Other	Total (%)
F	61	25	1	11	0	2	13
M	7	76	1	14	2	1	64
N	3	43	17	14	21	1	23

These percentages are sampled to simulate *H*_2_.

**Table 10.** Posterior estimates for coefficients of two Bayesian regression models predicting *MI*(*C*; *G*)/*H*(*C*)—mutual information between plural class *C* and gender *G*, normalized by plural class entropy *H*(*C*)—under *H*_2_ (gender conditioning at the level observed for monosyllabic German nouns).

	Expts. 1–2				Expt. 3			
	Estimate	Est. Error	l-95% CI	u-95% CI	Estimate	Est. Error	l-95% CI	u-95% CI
Intercept	−0.66	0.01	−0.68	−0.64	−0.85	0.01	−0.87	−0.83
*H*(*C*)	0.02	0.01	0.01	0.03	0.04	0.01	0.03	0.05
*φ*	22.60	0.10	22.41	22.79	13.40	0.06	13.29	13.52

#### Results.

**Figure 11.**
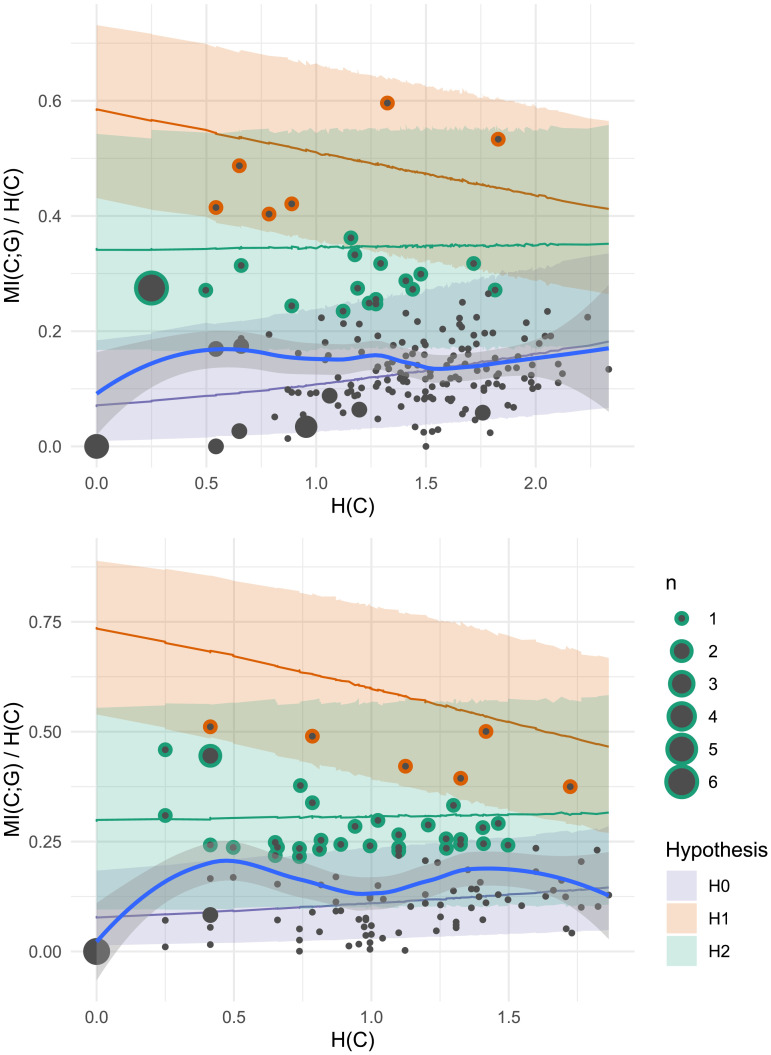
Plural class entropy *H*(*C*) and mutual information with grammatical gender *MI*(*C*; *G*) for each individual participant (dot) in Experiments 1–2 (upper) and 3 (lower). Shaded areas represent 95% confidence intervals under a) Bayesian regression models for *H*_1_ (gender conditioning for the overall lexicon; orange), *H*_2_ (gender conditioning for monosyllabic nouns; green) and *H*_0_ (null hypothesis of chance-level gender conditioning; purple) and b) a non-parametric Loess smoothed fit to the participant data (grey with blue line). Most participants are best fit by *H*_0_; the exceptions (15% in Expts. 1–2, 32% in Expt. 3) have outline colors indicating the best fit hypothesis based on Bayes Factor analysis.

Across experiments, the null hypothesis *H*_0_ remains the best fit to the data overall (Table 11). *H*_2_ has stronger statistical support than *H*_1_: 22 speakers (11% of the total) in Experiments 1 and 2, and 32 speakers (27%) in Experiment 3, have higher Bayes Factors for *H*_2_ than either other hypothesis—compare to 6 speakers in each experiment best fit by *H*_1_ (Figure 11). This suggests that some German speakers *do* use grammatical gender to predict plural class, but of those speakers, most treat gender as a second-order cue mediated by phonology rather than a first-order cue. Nonetheless, *H*_0_ is the best fit for decisive majorities in each experiment (85% in Experiments 1–2, 68% in Experiment 2). This means that, rather than match the lower level of gender conditioning found on similar word shapes in the lexicon, most German speakers instead appear to completely ignore grammatical gender in plural production.

**Table 11.** Bayes factor analysis for $\frac{H_{1}}{H_{0}}$ (*BF*_10_), $\frac{H_{2}}{H_{1}}$ (*BF*_21_), and $\frac{H_{2}}{H_{0}}$ (*BF*_20_).

Experiment	*BF*_10_	*BF*_21_	*BF*_20_
1–2	0	∞	0
3	0	∞	0

We find strongest support for the null hypothesis *H*_0_, corresponding to no gender conditioning (*MI*_*independent*_), followed by *H*_2_ (*MI*_*mediated*_), then *H*_1_ (*MI*_*overall*_).

### Orthographic Neighborhoods

*H*_2_ hypothesizes that coarse-grained phonology, i.e., word shape (expressed as syllable count), may mediate gender conditioning, and finds limited support—our experimental results show even lower levels of gender conditioning, more consistent with the null hypothesis *H*_0_, which disregards gender entirely. It remains possible, however, that finer-grained segmental phonological analysis would reveal a different picture. For example, if we consider the set of existing nouns that share segmental patterns with our stimuli, we may find an even weaker influence of grammatical gender among these nouns. This would suggest that fine-grained phonological properties of our stimuli lead speakers to disregard grammatical gender, consistent with our experimental observations. To address this consideration, we analyze the orthographic neighborhoods of our experimental stimuli (Table 1).

Milin et al. (2011) and Blevins et al. (2017) use lexical neighbors to predict inflection generalization in similar experimental settings. We use their approach to identify nearest neighbors for our novel German noun stimuli. For each stimulus item, we calculate its Levenshtein edit distance to each other noun in CELEX. We then normalize the edit distance by word length, and take as neighbors all words with a normalized Levenshtein distance less than or equal to .5.

**Figure 12.**
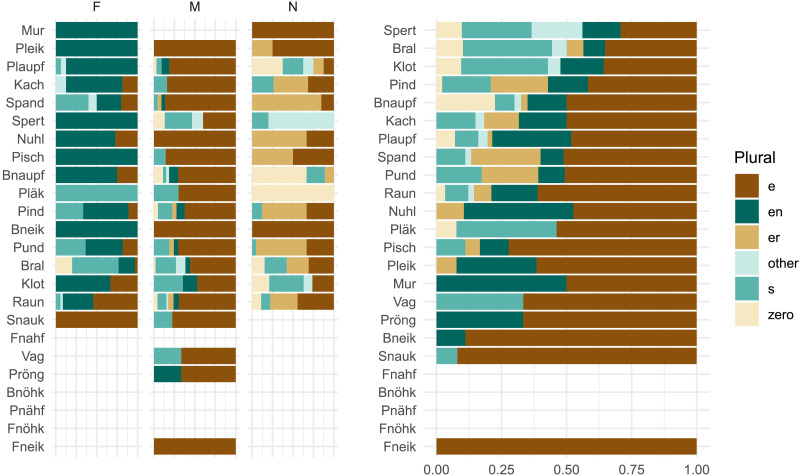
Distribution of plural classes for orthographic neighbors of the experimental stimuli. Items are ordered based on descending gender conditioning *MI*(*C*; *G*) (left) and overall plural class variation *H*(*C*) (right). Blank items have no words in CELEX which meet our neighbor criterion. For stimulus items with neighbors, grammatical gender explains on average (i.e., mean) 41% of the plural class variation in orthographic neighbors.

We find that four of the twenty-four items of our stimuli (*Fnahf*, *Bnöhk*, *Pnähf*, and *Fnöhk*) have no orthographic neighbors in CELEX. This is not surprising, as these four items are part of the twelve “Non-Rhymes” originally developed to test morphological generalization to phonologically atypical words (Marcus et al., 1995). Of the stimuli with neighbors, we observe a wide range of values for gender conditioning (Figure 12, left). For instance, grammatical gender perfectly predicts the plural class of orthographic neighbors for *Mur*, the item with the highest level of gender conditioning in Experiment 3. On the other hand, grammatical gender predicts none of the plural class variation in neighbors of *Vag* and *Bnöhk*. Segmental phonology clearly contributes to the generalization patterns observed in our experiments. It does not, however, explain the generally low level of gender conditioning. Among orthographic neighbors, grammatical gender accounts for about 41% of plural class variation on average—an even higher level of conditioning than found in the overall lexicon.

### Neural Network Model

The preceding analyses assess two potential phonological influences (word shape, segmental neighbors) on gender conditioning in plural class production, and find that neither one accounts for the low levels of gender conditioning observed in our three behavioral experiments. Nonetheless, it remains possible that these limited models fail to represent more nuanced interactions between phonological properties, and these rich phonological interactions in turn mediate grammatical gender’s utility as a predictor of plural class. In other words, a more powerful, flexible model, such as a neural network, may also learn to apply the same gender insensitivity to these stimuli.

In this final analysis, we compare recurrent neural networks (RNNs) trained with and without exposure to grammatical gender, to determine which better predicts speaker behavior. Conceptually, the RNN trained *without* gender may be viewed as a more sophisticated analog to *H*_0_, with the capacity to condition on the nouns’ orthographic form. The RNN trained *with* gender is less constrained: it may learn to rely on grammatical gender as expressed in *H*_1_, subordinate gender conditioning to phonology as in *H*_2_, or largely disregard gender as in *H*_0_. If the RNN trained with gender learns majority speaker-like plural production—i.e., patterning most closely with *H*_0_, and least with *H*_1_—this would indicate that grammatical gender’s diminished predictive role is straightforwardly learnable from its joint distribution with orthographic forms in the German noun lexicon. In this case, our experimental findings would not be so mysterious after all.

#### Procedure.

A neural encoder-decoder model encodes an input sequence into a fixed vector representation and then incrementally decodes it into a corresponding output sequence. We follow other recent work in using the recurrent neural architecture of Kann and Schütze (2016), which has been proposed for cognitive modeling (Kirov & Cotterell, 2018). For the task of German number inflection, the model takes as input a character sequence representing the singular nominative form of a noun, preceded by a special character for grammatical gender (e.g., 〈*f*〉 w a h l; 〈*f*〉 indicates feminine, 〈*m*〉 masculine, and 〈*n*〉 neuter). The model is trained to produce the noun’s corresponding nominative plural form as output (e.g., w a h l e n). We use the 11,243 German nouns in UniMorph version 1 (Kirov et al., 2016), and include noun gender by merging the dataset with another Wiktionary scrape.^13^ We divide the dataset into training (8694 nouns), development (1229), and test (1320) splits. We train two different versions of the model: one exposed to both noun gender and orthography, and one which sees only orthography.

Following Corkery et al. (2019), we train 25 separate random initializations of the same model architecture, with the same hyperparameters used by Kann and Schütze (2016). This allows separate model instances to be treated as simulated “speakers,” letting us aggregate productions and compare more directly to human speaker data. We train each instance for 10 epochs, achieving an average of 99% accuracy on the training set, and 88% on the test set—performance comparable to similar computational studies (e.g., Dankers et al., 2021; Goebel & Indefrey, 2000; Nakisa & Hahn, 1996).

#### Evaluation.

For the RNNs trained with gender, we combine each of the 24 noun stimuli with each of the three grammatical genders, and provide the resulting 72 items as input to each model instance. Following the “simulated speaker” approach, we compute *H*(*C*) and *MI*(*C*; *G*) for the productions of each RNN model instance trained with gender, and compute their log likelihood under each hypothesis—this lets us measure the extent to which the model matches speaker gender conditioning behavior.

For the models trained without gender, we provide only the 24 input forms. Here, *MI*(*C*; *G*) is zero by design, so we take a different approach to evaluation. We aggregate the productions of the 25 instances to produce a distribution of plural classes for each item and gender combination, and compare this to the distribution produced by speakers for that same item. In lieu of calculating log-likelihood as for the Bayesian models, we instead compute the Kullback-Leibler (KL) divergence (Lin, 1991) for each item:

$$D_{\text{KL}}\left(P∥Q\right)=\sum_{x\in 𝒳}P\left(x\right)\;\log\frac{P\left(x\right)}{Q\left(x\right)}$$ (14)

We treat the neural models’ item-level plural class predictions as the reference distribution *P*, and compute the divergence to speakers’ plural class productions in each experiment. In this analysis, we take the model with the *lowest* overall KL divergence as best approximating speaker productions—allowing us to directly compare the form-only and form-plus-gender RNN predictions.

#### Results.

The 25 instances of RNNs trained with grammatical gender show a range of gender conditioning behavior (Figure 14): 4 are best fit by the null hypothesis *H*_0_, 2–4 (depending on experimental design) align with the overall gender conditioning hypothesis *H*_1_, and the remaining 17–19 instances have the highest log likelihood under the mediation hypothesis *H*_2_. This suggests that the RNN implicitly learns the cue hierarchy expressed by *H*_2_, in which grammatical gender’s capacity to predict plural class is subordinate, and phonological cues such as word shape take precedence. Importantly, however, the RNN does not reproduce the same level of gender insensitivity shown by the majority of speakers in our experiments, whose behavior is most consistent with the null hypothesis *H*_0_.

**Figure 13.**
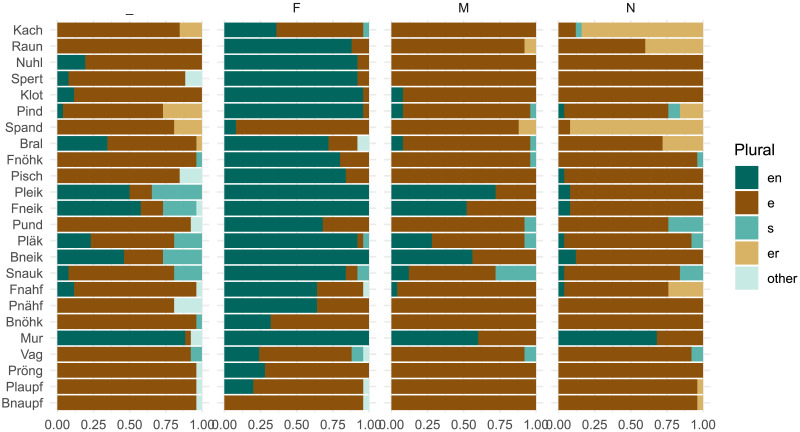
Item-level predictions for RNNs trained on orthographic form only (left column) and on orthographic form with gender (right three columns). Items are ordered by descending *MI*(*C*; *G*) in predictions from the form-plus-gender RNN.

Even though the RNN does not consistently match *MI*_*overall*_(*H*_0_), the level of gender conditioning observed in the overall lexicon, matching *MI*_*mediated*_(*H*_2_) nevertheless suffices to produce highly gender-conditioned plural class distributions at the item level (Figure 13; compare to the speaker productions in Figures 3 and 8). As a result, the RNNs with and without gender can vary substantially in their predicted item-level distributions. In terms of KL divergence from the plural distributions produced by speakers (Figure 15), neither model shows a strong advantage, but the form-only RNN appears closer on average—it shows a lower mean divergence for Experiments 1 and 2, and lower median divergence for all experiments. If anything, exposure to grammatical gender appears to slightly harm the RNN’s ability to model speaker-like plural production.

**Figure 14.**
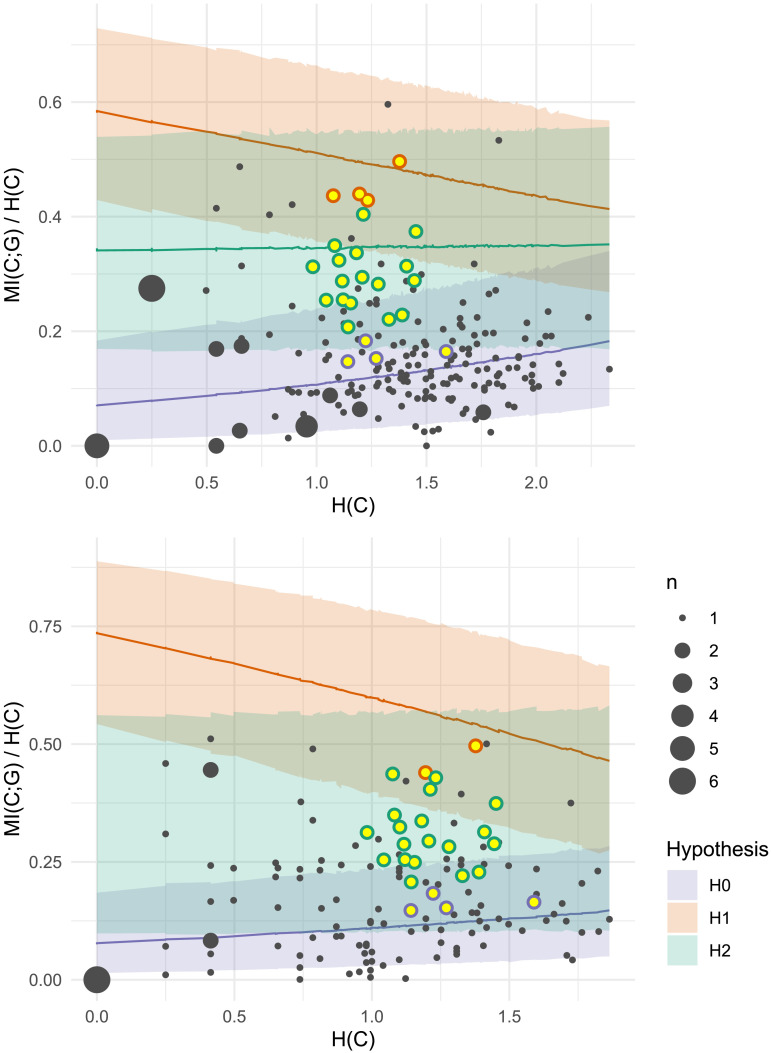
Plural class entropy *H*(*C*) and mutual information with grammatical gender *MI*(*C*; *G*) for each individual participant (grey dot) and RNN trained on noun form and gender (yellow dot) in Experiments 1–2 (left) and 3 (right). Shaded areas represent 95% confidence intervals under Bayesian regression models for *H*_1_ (gender conditioning for the overall lexicon; orange), *H*_2_ (gender conditioning for monosyllabic nouns; green) and *H*_0_ (null hypothesis of chance-level gender conditioning; purple). For RNN predictions, stroke color indicates best fit hypothesis. Note that the same 25 RNN predictions are plotted in both panels. Whereas most participants are best fit by *H*_0_, most RNN model predictions are best fit by *H*_2_.

**Figure 15.**
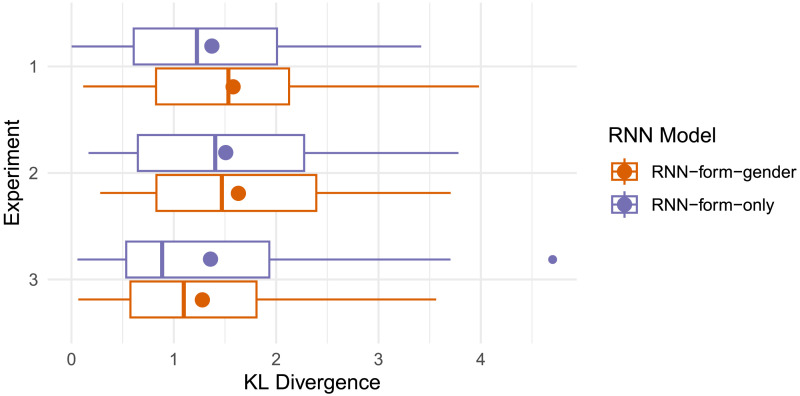
Item level KL divergence (lower is better) from RNN predictions to speaker plural productions in Experiments 1–3. Box shows 25%–75% percentiles, middle line shows median, dot shows mean. The form-only RNN shows lower mean KL divergence than the form-plus-gender RNN for Experiments 1 and 2, and lower median KL divergence for all three experiments.

## DISCUSSION

**Figure 16.**
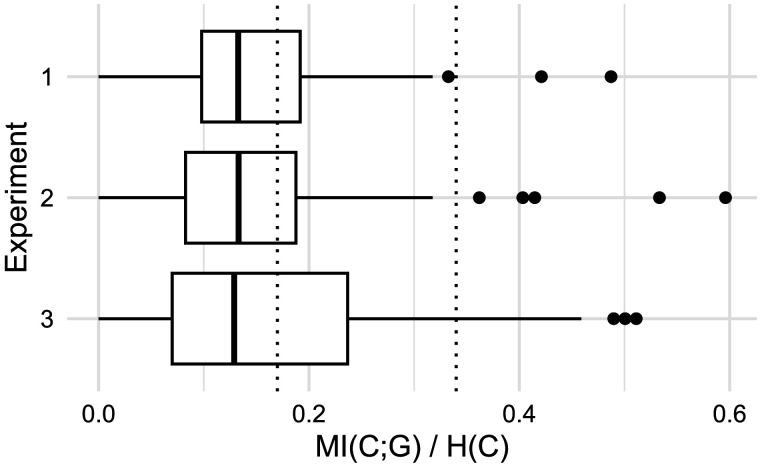
Distribution of gender conditioning, i.e., mutual information between plural class and grammatical gender *MI*(*C*; *G*) normalized by plural class entropy *H*(*C*), per participant per experiment. Boxplot shows median value (center line), 25%–75% quartiles (box edges), and 95% quartiles (lines). For comparison, dotted lines show *MI*(*C*; *G*)/*H*(*C*) for the German noun lexicon overall (34%) and for only monosyllabic nouns (17%). In each experiment, the median speaker conditions about 13% of plural class variation on grammatical gender.

The three experiments presented here point toward one consistent result: although grammatical gender predicts plural class in the German noun lexicon, the median adult German speaker ignores this cue when generalizing plural classes to unknown nouns. Figure 16 visualizes this gap. In each experiment, gender conditioning accounts for roughly 13% of variation in the median speaker’s plural class productions. This is considerably less than in the overall lexicon (34%), but also considerably more than zero, and not too far from the gender conditioning level observed on monosyllabic nouns like our stimuli (17%). To affirmatively claim that the median speaker ignores gender, we rely upon our hypothesis testing procedure. Across Figures 5, 6, 9, and 11, the blue regression line consistently falls within the confidence intervals of our null hypothesis model *H*_0_. As this model has been trained on simulated data where gender and plural class were independently sampled, we can confidently state that the median speaker’s behavior is equivalent to a model that ignores gender by design. Moreover, across all experiments, decisive majorities of speakers (65%+) show gender conditioning behavior which is more compatible with the null hypothesis *H*_0_ than either the overall gender conditioning model *H*_1_ or the phonologically mediated gender conditioning model *H*_2_.

A limitation of our study is that our stimuli only comprise monosyllables, meaning they cover a restricted range of the space of possible phonological forms of German nouns. Phonological cues are known to affect noun class learning broadly (Culbertson et al., 2017) and German plural inflection specifically (Köpcke, 1988; Mugdan, 1977; Spreng, 2004). Recent work by Monakhov et al. (2026) further supports a strong role for phonology in German plural class identification: they found that a network model of phonological sequence chunking successfully modeled participant behavior in guessing the plural class of existing German nouns when incrementally revealed letter-by-letter.^14^ Considering our results, several consistent item-specific patterns can be traced back to phonological overlap with a frequent German noun, such as the feminine *Mur* discussed in Experiment 3, or the disproportionate use of rare plural *-er* for *Pind* (presumably influenced by the frequent noun *Kind* ‘child’). The skewed gender assignments in Experiment 3 also likely reflect phonology; our noun stimuli appear more phonologically similar to nonfeminine nouns. Nevertheless, our analysis shows that phonology cannot fully account for the gender insensitivity shown by speakers in our experiments. It is true that grammatical gender is less predictive for German nouns which share phonological properties (e.g., word shape) with our stimuli. Our modeling shows, however, that even if phonological cues mediate gender conditioning for our stimuli, there should still be a stronger influence of gender than we find in our experimental results.

Another takeaway from our findings is the need to analyze variation at the level of individual speakers. As we saw in Experiment 1, standard null hypothesis significance testing indicates a robust effect of grammatical gender on plural class production. Our speaker-level analysis, however, reveals that this effect is driven by a minority—fewer than 35% of speakers in each experiment. By simulating expected speaker behavior under different hypotheses, we operationalize the null hypothesis in a Bayesian framework and demonstrate that it better fits the production data of the median German speaker.

One possible account of speakers’ gender insensitivity is in terms of cue *blocking*.^15^ In the discriminative learning literature (e.g., Ramscar, 2023), blocking reflects cue sequencing: if a speaker first learns to associate a particular outcome (here, plural class) with a particular cue or set of cues (here, phonology), this could block them from learning to associate that same outcome with another cue (here, grammatical gender). Phonology provides the earliest set of cues available to language learners, and the blocking account is consistent with findings that children privilege phonological cues in acquisition (Culbertson et al., 2019; Szagun et al., 2007). The key question here is whether such cue blocking should persist into adulthood, as adults have been found to generalize based on semantic cues where children instead rely on phonology in both artificial (Pertsova & Becker, 2021) and natural (Gagliardi & Lidz, 2014) language experiments. Moreover, our results show that a minority of German speakers indeed reproduce levels of gender conditioning found in the overall lexicon. Discriminative models of German number inflection (e.g., Heitmeier et al., 2021) might further specify how gender and phonological cues interact under this account.

Finally, our findings raise a critical question: how does the lexical relationship between gender and plural class persist, if the majority of German speakers don’t extend it to novel words? This disconnect between linguistic structure and speaker behavior is puzzling, but the language evolution literature suggests one possible explanation. In an artificial language learning experiment iterated over populations, Smith and Wonnacott (2010) found that most speakers probability-matched the distribution of noun classes—but if one speaker introduced a structural regularity such as lexical conditioning, later generations of speakers could maintain and spread that conditional relationship. Subsequent work has shown that linguistic structure is shaped by inductive biases in *cultural transmission*, which often diverge from the inductive biases of individual speakers (Ferdinand et al., 2019; Smith et al., 2017). The gender-plural relation may reflect cultural transmission, while individual speaker generalization may reflect other factors such as phonological bias. Future behavioral and computational research may someday explain the complex interaction between the structured, social, symbolic systems of natural language, and the cognitive processes of individual speakers through which those systems are realized.

## CONCLUSION

Many aspects of language production straightforwardly reflect linguistic distributions, as speakers use predictive cues to help select between possible linguistic forms. We study the well-established conditional relationship between one such predictor—the grammatical gender of German nouns—and a variable target category—the plural class of those same nouns. Although the distributional evidence for this relationship is robust, we find across three experiments that the median German speaker ignores grammatical gender when generalizing plural forms to unknown nouns. Our results highlight an underexplored direction in cognitive science: accounting for the linguistic information that speakers *don’t* use. As powerful statistical models of linguistic distributions reshape the scientific landscape, we anticipate a growing need to identify and theorize cases like this one.

## Notes

^1^ “Speaker” is used throughout this paper in the more general sense of “language user,” with no intended restriction to oral or auditory language use.^2^ The code and data supporting this analysis are released in the supplementary materials.^3^ We here follow the literature in focusing on the nominative-case form of each noun, considered the citation form.^4^ The gender distinction on the article is collapsed for plural inflection: all plural nouns take the definite article *die*.^5^ For comparison, a *prima facie* irrelevant conditioning variable such as “the noun contains the letter *a*, the letter *i*, both, or neither” has 0.02 bits of mutual information with plural class, corresponding to 1% of observed variation.^6^ Translation by the first author, emphasis in original.^7^ Note that our post-hoc analysis introduces a third hypothesis to model the influence of phonology. For clarity, we consider only *H*_0_ and *H*_1_ while reporting our experimental findings, and bring in the third hypothesis to analyze the combined results.^8^ Participants were recruited through the platform Prolific. Of 100 tested, 8 were excluded for failing attention checks.^9^ We use the lme4 library (Bates et al., 2015).Formula: suffix = = ["en"/"e"] ∼ Gender + (1|Participant) + (1|Item)^10^ Zaretsky and Lange perform multiple analyses with a range of other predictors, but we focus here on gender for simplicity.^11^ We thank the editor for suggesting this analysis. We also attempted per-item random slopes, but in that case the models failed to converge.Formula: suffix = = ["en"/"e"] ∼ Gender + (Gender|Participant) + (1|Item)^12^ In practice, orthography stands in for phonology in our analysis, consistent with the written modality of our behavioral experiments.^13^ https://github.com/gambolputty/german-nouns; cf. Dankers et al. (2021).^14^ We note that their task omits grammatical gender entirely, compelling participants to rely solely on phonology.^15^ We thank an anonymous reviewer for suggesting this interpretation of our results.

## References

[bib1] Arik, E. (2015). An experimental study of Turkish Vowel Harmony. Poznan Studies in Contemporary Linguistics, 51(3), 359–374. 10.1515/psicl-2015-0014

[bib2] Augst, G. (1979). Neuere forschungen zur substantivflexion. Zeitschrift für Germanistische Linguistik, 7, 220–232.

[bib3] Baayen, R. H., Piepenbrock, R., & Gulikers, L. (1995). The CELEX lexical database (release 2) [CD-ROM]. Linguistic Data Consortium, University of Pennsylvania.

[bib4] Bates, D., Mächler, M., Bolker, B., & Walker, S. (2015). Fitting linear mixed-effects models using lme4. Journal of Statistical Software, 67(1), 1–48. 10.18637/jss.v067.i01

[bib5] Becker, M., Ketrez, N., & Nevins, A. (2011). The surfeit of the stimulus: Analytic biases filter lexical statistics in Turkish laryngeal alternations. Language, 87(1), 84–125. 10.1353/lan.2011.0016

[bib6] Beser, D. (2021). Falling through the gaps: Neural architectures as models of morphological rule learning. In W. T. Fitch, C. Lamm, H. Leder, & K. Teßmar-Raible (Eds.), Proceedings of the 43rd Annual Conference of the Cognitive Science Society (pp. 1042–1048). Cognitive Science Society.

[bib7] Bittner, D. (1994). Die bedeutung der genusklassifikation für die organisation der deutschen substantivflexion. In K.-M. Köpcke (Ed.), Funktionale untersuchungen zur deutschen nominal- und verbalmorphologie (pp. 65–80). Niemeyer. 10.1515/9783111339825-005

[bib8] Bittner, D. (2000). Gender classification and the inflectional system of German nouns. In B. Unterbeck, M. Rissanen, T. Nevalainen, & M. Saari (Eds.), Gender in grammar and cognition: I: Approaches to gender. II: Manifestations of gender (pp. 1–24). De Gruyter Mouton. 10.1515/9783110802603.1

[bib9] Blevins, J. P., Milin, P., & Ramscar, M. (2017). The Zipfian paradigm cell filling problem. In F. Kiefer, J. P. Blevins, & H. Bartos (Eds.), Perspectives on morphological structure: Data and analyses (pp. 139–158). Brill. 10.1163/9789004342934_008

[bib10] Bloomfield, L. (1933). Language. Motilal Banarsidass Publishing House. (reissued edition 1994)

[bib11] Bürkner, P.-C. (2017). brms: An R package for Bayesian multilevel models using Stan. Journal of Statistical Software, 80(1), 1–28. 10.18637/jss.v080.i01

[bib14] Corkery, M., Matusevych, Y., & Goldwater, S. (2019). Are we there yet? Encoder-decoder neural networks as cognitive models of English past tense inflection. In A. Korhonen, D. Traum, & L. Màrquez (Eds.), Proceedings of the 57th Annual Conference of the Association for Computational Linguistics (pp. 3868–3877). Association for Computational Linguistics. 10.18653/v1/P19-1376

[bib15] Culbertson, J., Gagliardi, A., & Smith, K. (2017). Competition between phonological and semantic cues in noun class learning. Journal of Memory and Language, 92, 343–358. 10.1016/j.jml.2016.08.001

[bib16] Culbertson, J., Jarvinen, H., Haggarty, F., & Smith, K. (2019). Children’s sensitivity to phonological and semantic cues during noun class learning: Evidence for a phonological bias. Language, 95(2), 268–293. 10.1353/lan.2019.0031

[bib17] Dankers, V., Langedijk, A., McCurdy, K., Williams, A., & Hupkes, D. (2021). Generalising to German plural noun classes, from the perspective of a recurrent neural network. In A. Bisazza & O. Abend (Eds.), Proceedings of the 25th Conference on Computational Natural Language Learning (pp. 94–108). Association for Computational Linguistics. 10.18653/v1/2021.conll-1.8

[bib19] Elgersma, D., & Houseman, P. (1999). Optimality theory and natural morphology: An analysis of German plural formation. Folia Linguistica, 33(3–4), 333–354. 10.1515/flin.1999.33.3-4.333

[bib20] Ferdinand, V., Kirby, S., & Smith, K. (2019). The cognitive roots of regularization in language. Cognition, 184, 53–68. 10.1016/j.cognition.2018.12.002, 30572180

[bib21] Gaeta, L. (2008). Die deutsche pluralbildung zwischen deskriptiver angemessenheit und sprachtheorie. Zeitschrift für Germanistische Linguistik, 36(1), 74–108. 10.1515/ZGL.2008.005

[bib22] Gagliardi, A., & Lidz, J. (2014). Statistical insensitivity in the acquisition of Tsez noun classes. Language, 90(1), 58–89. 10.1353/lan.2014.0013

[bib23] Gawlitzek-Maiwald, I. (1994). How do children cope with variation in the input? The case of German plurals and compounding. In R. Tracy & E. Lattey (Eds.), How tolerant is universal grammar? Essays on language learnability and language variation (pp. 225–266). Niemeyer. 10.1515/9783111634777.225

[bib25] Goebel, R. & Indefrey, P. (2000). A recurrent network with short-term memory capacity learning the German *-s* plural. In P. Broeder & J. Murre (Eds.), Models of language acquisition: Inductive and deductive approaches (pp. 177–200). Oxford University Press. 10.1093/oso/9780198299899.003.0009

[bib26] Hahn, U., & Nakisa, R. C. (2000). German inflection: Single route or dual route? Cognitive Psychology, 41(4), 313–360. 10.1006/cogp.2000.0737, 11121259

[bib27] Hayes, B., & Londe, Z. C. (2006). Stochastic phonological knowledge: The case of Hungarian vowel harmony. Phonology, 23(1), 59–104. 10.1017/S0952675706000765

[bib28] Hayes, B., Zuraw, K., Siptár, P., & Londe, Z. (2009). Natural and unnatural constraints in Hungarian vowel harmony. Language, 85(4), 822–863. 10.1353/lan.0.0169

[bib29] Heitmeier, M., Chuang, Y.-Y., & Baayen, R. H. (2021). Modeling morphology with linear discriminative learning: Considerations and design choices. Frontiers in Psychology, 12, 720713. 10.3389/fpsyg.2021.720713, 34867600 PMC8634146

[bib30] Hudson Kam, C. L., & Newport, E. L. (2005). Regularizing unpredictable variation: The roles of adult and child learners in language formation and change. Language Learning and Development, 1(2), 151–195. 10.1080/15475441.2005.9684215

[bib31] Hudson Kam, C. L., & Newport, E. L. (2009). Getting it right by getting it wrong: When learners change languages. Cognitive Psychology, 59(1), 30–66. 10.1016/j.cogpsych.2009.01.001, 19324332 PMC2703698

[bib32] Indefrey, P. (1999). Some problems with the lexical status of nondefault inflection. Behavioral and Brain Sciences, 22(6), 1025. 10.1017/S0140525X99342229

[bib34] Kann, K., & Schütze, H. (2016). Single-model encoder-decoder with explicit morphological representation for reinflection. In K. Erk & N. A. Smith (Eds.), Proceedings of the 54th Annual Meeting of the Association for Computational Linguistics (Volume 2: Short Papers) (pp. 555–560). Association for Computational Linguistics. 10.18653/v1/P16-2090

[bib35] Kirov, C., & Cotterell, R. (2018). Recurrent neural networks in linguistic theory: Revisiting Pinker and Prince (1988) and the past tense debate. Transactions of the Association for Computational Linguistics, 6, 651–665. 10.1162/tacl_a_00247

[bib36] Kirov, C., Sylak-Glassman, J., Que, R., & Yarowsky, D. (2016). Very-large scale parsing and normalization of Wiktionary morphological paradigms. In N. Calzolari, K. Choukri, T. Declerck, S. Goggi, M. Grobelnik, B. Maegaard, J. Mariani, H. Mazo, A. Moreno, J. Odijk, & S. Piperidis (Eds.), Proceedings of the Tenth International Conference on Language Resources and Evaluation (LREC 2016) (pp. 3121–3126). European Language Resources Association (ELRA).

[bib37] Ković, V., Westermann, G., & Plunkett, K. (2008). Implicit vs. explicit learning in German noun plurals. Psihologija, 41(4), 387–411. 10.2298/PSI0804387K

[bib38] Köpcke, K.-M. (1988). Schemas in German plural formation. Lingua, 74(4), 303–335. 10.1016/0024-3841(88)90064-2

[bib39] Lin, J. (1991). Divergence measures based on the Shannon entropy. IEEE Transactions on Information Theory, 37(1), 145–151. 10.1109/18.61115

[bib40] Mahowald, K., Ivanova, A. A., Blank, I. A., Kanwisher, N., Tenenbaum, J. B., & Fedorenko, E. (2024). Dissociating language and thought in large language models. Trends in Cognitive Sciences, 28(6), 517–540. 10.1016/j.tics.2024.01.011, 38508911 PMC11416727

[bib41] Marcus, G. F., Brinkmann, U., Clahsen, H., Wiese, R., & Pinker, S. (1995). German inflection: The exception that proves the rule. Cognitive Psychology, 29(3), 189–256. 10.1006/cogp.1995.1015, 8556846

[bib43] Massey, J. L. (1994). Guessing and entropy. In Proceedings of 1994 IEEE International Symposium on Information Theory (p. 204). IEEE. 10.1109/ISIT.1994.394764

[bib44] McCurdy, K., Goldwater, S., & Lopez, A. (2020). Inflecting when there’s no majority: Limitations of encoder-decoder neural networks as cognitive models for German plurals. In D. Jurafsky, J. Chai, N. Schluter, & J. Tetreault (Eds.), Proceedings of the 58th Annual Meeting of the Association for Computational Linguistics (pp. 1745–1756). Association for Computational Linguistics. 10.18653/v1/2020.acl-main.159

[bib45] Milin, P., Keuleers, E., & Đurđević, D. (2011). Allomorphic responses in Serbian pseudo-nouns as a result of analogical learning. Acta Linguistica Hungarica, 58(1–2), 65–84. 10.1556/ALing.58.2011.1-2.4

[bib46] Miller, G. A., & Nicely, P. E. (1955). An analysis of perceptual confusions among some English consonants. Journal of the Acoustical Society of America, 27(2), 338–352. 10.1121/1.1907526

[bib48] Misersky, J., Majid, A., & Snijders, T. M. (2019). Grammatical gender in German influences how role-nouns are interpreted: Evidence from ERPs. Discourse Processes, 56(8), 643–654. 10.1080/0163853X.2018.1541382

[bib49] Monakhov, S., Diessel, H., & Balthes, B. (2026). Learn what is detectable, detect what is useful: Acquisition of German plural as a classification problem. Cognition, 266, 106292. 10.1016/j.cognition.2025.106292, 40882586

[bib50] Mugdan, J. (1977). Flexionsmorphologie und psycholinguistik: Untersuchungen zu sprachlichen regeln und ihrer beherrschung durch aphatiker, kinder und ausländer, am beispiel der deutschen substantivdeklination (Vol. 82). TBL-Verlag Narr.

[bib51] Nakisa, R. C., & Hahn, U. (1996). Where defaults don’t help: The case of the German plural system. arXiv. 10.48550/arXiv.cmp-lg/9605020

[bib52] Perfors, A. (2016). Adult regularization of inconsistent input depends on pragmatic factors. Language Learning and Development, 12(2), 138–155. 10.1080/15475441.2015.1052449

[bib53] Pertsova, K., & Becker, M. (2021). In support of phonological bias in implicit learning. Language Learning and Development, 17(2), 128–157. 10.1080/15475441.2020.1802279

[bib54] Pierrehumbert, J. B. (2022). More than seventy years of probabilistic phonology. In B. E. Dresher & H. van der Hulst (Eds.), The Oxford history of phonology (pp. 639–655). Oxford University Press. 10.1093/oso/9780198796800.003.0030

[bib55] Plag, I., Heitmeier, M., & Domahs, F. (2023). German nominal number interpretation in an impaired mental lexicon: A naive discriminative learning perspective. The Mental Lexicon, 18(3), 417–445. 10.1075/ml.23017.pla

[bib56] R Core Team. (2023). R: A language and environment for statistical computing. R Foundation for Statistical Computing. https://www.R-project.org/

[bib57] Ramscar, M. (2023). A discriminative account of the learning, representation and processing of inflection systems. Language, Cognition and Neuroscience, 38(4), 446–470. 10.1080/23273798.2021.2014062

[bib58] Regel, S., Opitz, A., Müller, G., & Friederici, A. D. (2019). Processing inflectional morphology: ERP evidence for decomposition of complex words according to the affix structure. Cortex, 116, 143–153. 10.1016/j.cortex.2018.10.003, 30466728

[bib59] Schuhmann, K. S., & Putnam, M. T. (2021). Relativized prosodic domains: A late-insertion account of German plurals. Languages, 6(3), 142. 10.3390/languages6030142

[bib60] Shanks, D. R., Tunney, R. J., & McCarthy, J. D. (2002). A re-examination of probability matching and rational choice. Journal of Behavioral Decision Making, 15(3), 233–250. 10.1002/bdm.413

[bib61] Shannon, C. E. (1948). A mathematical theory of communication. The Bell System Technical Journal, 27(3), 379–423. 10.1002/j.1538-7305.1948.tb01338.x

[bib62] Smith, K., Perfors, A., Fehér, O., Samara, A., Swoboda, K., & Wonnacott, E. (2017). Language learning, language use and the evolution of linguistic variation. Philosophical Transactions of the Royal Society B: Biological Sciences, 372(1711), 20160051. 10.1098/rstb.2016.0051, 27872370 PMC5124077

[bib63] Smith, K., & Wonnacott, E. (2010). Eliminating unpredictable variation through iterated learning. Cognition, 116(3), 444–449. 10.1016/j.cognition.2010.06.004, 20615499

[bib64] Spreng, B. (2004). Error patterns in the acquisition of German plural morphology: Evidence for the relevance of grammatical gender as a cue. Toronto Working Papers in Linguistics, 23(2), 147–172.

[bib65] Szagun, G., Stumper, B., Sondag, N., & Franik, M. (2007). The acquisition of gender marking by young German-speaking children: Evidence for learning guided by phonological regularities. Journal of Child Language, 34(3), 445–471. 10.1017/S0305000906007951, 17822135

[bib66] Trommer, J. (2021). The subsegmental structure of German plural allomorphy. Natural Language & Linguistic Theory, 39(2), 601–656. 10.1007/s11049-020-09479-7

[bib68] Vulkan, N. (2000). An economist’s perspective on probability matching. Journal of Economic Surveys, 14(1), 101–118. 10.1111/1467-6419.00106

[bib69] Wegener, H. (1994). Variation in the acquisition of German plural morphology by second language learners. In R. Tracy & E. Lattey (Eds.), How tolerant is universal grammar? Essays on language learnability and language variation (pp. 267–294). Niemeyer. 10.1515/9783111634777.267

[bib70] Wiese, R. (1996). The phonology of German. Oxford University Press.

[bib71] Yang, C. (2016). The price of linguistic productivity: How children learn to break the rules of language. MIT Press. 10.7551/mitpress/9780262035323.001.0001

[bib72] Zaretsky, E., & Lange, B. P. (2016). No matter how hard we try: Still no default plural marker in nonce nouns in Modern High German. In H. Christ, D. Klenovšak, L. Sönning, & V. Werner (Eds.), A blend of MaLT: Selected contributions from the Methods and Linguistic Theories Symposium 2015 (pp. 153–178). University of Bamberg Press.

